# USE OF INFORMATION AND COMMUNICATION TECHNOLOGY AND MEMORY PERFORMANCE IN OLDER ADULTS: WHICH COMES FIRST?

**DOI:** 10.1093/geroni/igz038.1198

**Published:** 2019-11-08

**Authors:** Eun Young Choi, Elizabeth M Zelinski

**Affiliations:** Davis School of Gerontology, University of Southern California, Los Angeles, California, United States

## Abstract

The topic of older adults’ information and communication technology (ICT) use looms large because of the beneficial effects of ICT use on physical health, emotional well-being, and social engagement. Previous research has shown that memory performance is also linked with ICT use, but the direction of influence is yet to be determined. Individuals with higher levels of memory function are more likely to use ICT devices, but ICT use may have protective effects on maintaining memory because using technologies includes mental exercises. The current study examined the temporal sequence of ICT use and memory performance, which can provide insight into the causation. Using three waves (2013, 2015, and 2017) from the National Health and Aging Trends Study (NHATS), a total of 4,048 community-dwelling older adults aged 65 and above were selected for the analysis. Memory performance was measured by summing scores of immediate and delayed word recall. Reciprocal 5-year lagged associations between ICT use and memory were examined, while controlling for age, gender, education, racial/ethnic minority status, and depressive symptoms. The final model showed adequate fit indices (CFI = .979 and RMSEA = .038). Word recall significantly predicted ICT use in later years. Reciprocally, greater use of ICT was significantly associated with better memory performance in following years. The effect of ICT use on memory performance was of greater magnitude in comparison with memory as a predictor for ICT use. These results suggest that ICT can have potential benefits for maintaining memory in old age.

